# The Application of Artificial Intelligence in Acute Prescribing in Homeopathy: A Comparative Retrospective Study

**DOI:** 10.3390/healthcare13151923

**Published:** 2025-08-06

**Authors:** Rachael Doherty, Parker Pracjek, Christine D. Luketic, Denise Straiges, Alastair C. Gray

**Affiliations:** HOHM Foundation, Office of Research, Philadelphia, PA 19138, USA; research@hohmfoundation.org (R.D.); parker.pracjek@hohmfoundation.org (P.P.); christine.d.luketic@hohmfoundation.org (C.D.L.); denise.straiges@hohmfoundation.org (D.S.)

**Keywords:** homeopathy, artificial intelligence, large language models, clinical outcomes, teaching clinic, MYCaW

## Abstract

Background/Objective: The use of artificial intelligence to assist in medical applications is an emerging area of investigation and discussion. The researchers studied whether there was a difference between homeopathy guidance provided by artificial intelligence (AI) (automated) and live professional practitioners (live) for acute illnesses. Additionally, the study explored the practical challenges associated with validating AI tools used for homeopathy and sought to generate insights on the potential value and limitations of these tools in the management of acute health complaints. Method: Randomly selected cases at a homeopathy teaching clinic (*n* = 100) were entered into a commercially available homeopathic remedy finder to investigate the consistency between automated and live recommendations. Client symptoms, medical disclaimers, remedies, and posology were compared. The findings of this study show that the purpose-built homeopathic remedy finder is not a one-to-one replacement for a live practitioner. Result: In the 100 cases compared, the automated online remedy finder provided between 1 and 20 prioritized remedy recommendations for each complaint, leaving the user to make the final remedy decision based on how well their characteristic symptoms were covered by each potential remedy. The live practitioner-recommended remedy was included somewhere among the auto-mated results in 59% of the cases, appeared in the top three results in 37% of the cases, and was a top remedy match in 17% of the cases. There was no guidance for managing remedy responses found in live clinical settings. Conclusion: This study also highlights the challenge and importance of validating AI remedy recommendations against real cases. The automated remedy finder used covered 74 acute complaints. The live cases from the teaching clinic included 22 of the 74 complaints.

## 1. Introduction

### 1.1. Homeopathy

Homeopathic medicine is a whole system of health care and therapeutics developed over 200 years ago by Samuel Hahnemann, a German physician [1]. Although this principle of healing dated from Hippocrates [2], Hahnemann was the first to systematize it by testing and rigorously documenting symptoms caused by giving medicinal substances to healthy people [3].

One of the principal distinctions between homeopathy and conventional medicine is homeopathy’s focus on choosing a medicine based on an individual’s unique constellation of symptoms rather than the common symptoms of a named condition [1]. Although Hahnemann found and documented evidence with this approach, it was significantly more labor intensive than conventional methods, requiring a detailed knowledge of dozens of medicinal substances to match a sick person’s unique symptoms. Homeopaths developed sophisticated indexes to assist them in finding curative remedies for their patients from the earliest days because there were thousands of symptoms associated with each medicinal substance [4].

Modern-day homeopaths have access to software (sometimes described as repertory software) to help them match the symptoms of their clients to potential remedies [5]. Even with this resource, identifying a curative remedy can be a labor-intensive process—one that requires a detailed client intake, the ability to focus on multiple body systems at once, and the ability to distinguish between the common symptoms relied on in conventional medicine (e.g., an ear infection with a cough) and the more specific characteristic symptoms that are the pointers to potentially supportive homeopathic remedies (e.g., ear pain that feels better for wrapping up the head and a dry cough triggered by irritation in the larynx) [6,7,8].

### 1.2. AI in Medicine

Artificial intelligence is revolutionizing medicine in a number of ways. These ways include but are not limited to diagnostic imaging, chatbots, and computer-assisted decision-making for physicians [9,10]. While this is a rapidly developing field with possibilities for significant progress in easing the burden of human suffering there is also the potential for misunderstanding and misuse [11,12].

Despite the possible risks, developers, practitioners, and consumers alike continue to drive the development of AI tools for medicine. Developers see a profitable, growing industry already worth an estimated $13.7 billion and expected to grow to $156.8 billion by 2033 [13]. Practitioners see the potential of a more efficient use of their time as well as keeping up with technological advances [14]. Consumers may see benefits such as an increase in empowerment and support for mental health, and there is also a very real and growing problem in access to primary care that will likely continue the demand for AI tools in the regulated field of conventional health and medicine [12,15].

### 1.3. How AI Is Used in Homeopathy

The principal distinction between homeopathy and conventional medicine is homeopathy’s focus on choosing a medicine based on an individual’s unique constellation of symptoms rather than the common symptoms of a named condition [1]. Homeopaths have long relied on evidence provided in the literature—from homeopathic provings, case reports, or compilations of remedy profiles from experienced practitioners to guide remedy selection. A detailed knowledge of dozens of medicinal substances is difficult for any one practitioner to memorize. However, this knowledge is required to match a sick person’s individual symptoms. Homeopaths have developed sophisticated reference tools to assist them in finding curative remedies for their patients long before the arrival of the digital age [4].

Expertise is required to successfully manage a homeopathic case. This includes individual client intake, an understanding of the acute illness symptoms and listening to all of the client concerns. These skills do not easily transfer to AI, yet the profession may be well-positioned for the integration of AI tools into some work processes. These processes may include a focus on compiling the multiple types and formats of information available to align the characteristic symptoms of the sick person with the characteristic symptoms of the remedy. The automated remedy finder we used for this investigation, which focuses on acute complaints only, is not designed to mimic the rapport building or empathetic communication that is a critical element of chronic case taking.

Purpose-built online remedy finders of varying levels of sophistication and cost are available. At the time of our research, these included ABCHomeopathy.com [16], Homeopathic Housecall [17], and Remedeo [18]. These tools have the same potential attraction as other online conventional AI medical tools—the prospect of increased access to information to guide choices to improve the level of health of a sick person. However, little is known about how accurate these tools are, how the models are validated, and the degree to which they can replace the professional guidance of a live homeopath.

### 1.4. Current Questions

While purpose-built homeopathic remedy finders are designed to reflect remedy profiles available in the homeopathic literature, it is unknown whether the remedy recommendations provided by these tools are similar to recommendations given by live practitioners when presented with similar real-world symptoms. There are also questions about non-remedy-related recommendations from automated homeopathy tools, including the types of complaints the automated tools can effectively cover, what guidance is provided on remedy potency and dosage frequency, and when to seek a licensed health care provider.

This research presents the findings from a comparison of acute complaints managed by a live homeopathy teaching clinic to one commercially available, purpose-built AI homeopathic remedy finder’s recommendations generated for acute complaints. The comparison included the types of complaints covered, medical disclaimers, remedy recommendations, and posology (i.e., recommendations on remedy potency, frequency, and delivery method).

### 1.5. Aim

The primary aim of this study is to investigate the degree to which automated homeopathic remedy recommendations for acute complaints overlap with remedy recommendations from a live practitioner. For this preliminary inquiry, researchers chose one of several purpose-built remedy finders based on a methodological review of several similar tools. The tool chosen to investigate by this research team was based upon its commercial availability and simplicity of use for consumers, and that it is an electronic version of an 18th century initiative by the prominent American homeopath Constantine Hering. A secondary aim of this investigation was to better understand how basic case management issues are handled by an automated remedy finder. An additional aim was to generate spin-off research questions for further investigation in this field. The research illustrated how real data can be used to assess the accuracy of an automated tool as well as the inherent challenges and limitations of the process.

## 2. Materials and Methods

### 2.1. Study Design

This comparative retrospective study compared remedy recommendations for 100 previous acute clinic clients from the Academy of Homeopathy Education (AHE) [19] teaching clinic, part of HOHM Foundation [20] against a commercially available online remedy finder [17].

### 2.2. Procedures

Inclusion criteria were identified a priori to the research. There were 307 unique cases that met the initial inclusion criteria (described below) for this retrospective case comparison. An online random number generator from Calculator.net was utilized to randomize the pool of cases. Of those cases selected through randomization, each case was then individually assessed to determine whether at least one of the symptoms of the case matched a complaint category listed by the online remedy finder. Researchers used the information provided in the case notes to answer the online remedy finder’s automated questionnaire for the first assigned complaint. Answers to each question were logged as well as the remedy recommendations provided by the online tool. If one of the remedy recommendations provided by the online tool matched the remedy recommendation made by the live clinicians, the comparison was considered complete for that case. If there was no remedy match with the first complaint, researchers analyzed the case a second time using the second assigned complaint where possible. This occurred 25 times over the course of 100 cases analyzed. Researchers compared 125 discrete complaints between the online remedy finder and the clinician notes.

### 2.3. Participants

Clients come to the clinic at AHE in a number of ways. The client cases selected for this study were either self-referred, recommended by other clients, recommended by students at AHE, by other homeopaths or health practitioners, or from online homeopathy study groups. There were no demographic restrictions. Clients could be of any gender, age, or background. Clients had their cases taken either by AHE’s clinical instructors, or by advanced students under supervision. Consent for use of clinical data for research purposes was collected at the initial case intake. A total number of 119 acute cases were reviewed for inclusion. 19 cases were excluded based upon the a priori inclusion/exclusion criteria, leaving 100 cases as the study sample. There were 25 cases (25%) in which 2 distinct client complaints could be mapped to 2 distinct online remedy finder complaints, and 29 cases (29%) in which there was no remedy match with no alternate complaint available to run through the online program.

### 2.4. Inclusion and Exclusion Criteria

Acute case records were selected based on the following apriori criteria:

1. Any client of any age who sought homeopathic care for acute complaints at the AHE teaching clinic during the years 2022–2023.
2. Cases selected had complete case data and had at least 1 follow-up consultation.
3. Cases selected where the client reported compliance with taking the remedy.
4. Cases where initial and follow-up remedy response scores were complete.
5. Cases where initial and follow-up ‘Measure Yourself Concerns and Well-being’ (MYCaW) scale [21] scores were complete.

To be included in this study, a suitable predefined complaint category needed to neatly cover at least one of the MYCaW concerns. Cases were excluded if either MYCaW concern could not confidently be matched to a complaint category.

### 2.5. Data Analysis

Descriptive statistics were used to quantify the results of the comparisons between the AI tool and the clinic data. This was planned to emphasize the nature of the research being conducted. The research questions focused on a small sample of outcomes from a comparison between a practitioner lead clinic and an AI tool. The outcomes planned and measured were as follows:

1. Overlap between the clinic and online remedy recommendations.
2. Comparison of where the AHE-recommended remedy appeared in the prioritized list of remedies recommended by the online remedy finder.
3. Range and frequency of complaints used in the comparison to assess the accuracy of the findings.
4. The types of complaints for which there were top matches with the online remedy finder as well as the remedies used.

## 3. Results

### 3.1. Complaints Covered

The automated remedy finder covers a total of 74 complaints (see Appendix A), 16 of which are covered by medical disclaimers. The 16 complaints covered by medical disclaimers were:

1. Allergic reactions;
2. Bone fracture;
3. Burns;
4. Chest Pain;
5. Circulatory shock;
6. COVID-19;
7. Croup;
8. Drowning;
9. Fainting;
10. Frostbite;
11. Head injury/concussion;
12. Kidney stone;
13. Mumps;
14. Poisoning (food or alcohol);
15. Traumatic injuries;
16. Wounds to the skin.

### 3.2. Complaints Excluded

There were 100 cases identified in the random selection for the study. Of these, fifteen cases were excluded from because the acute complaints prioritized by the clinic clients in the MYCaW were not covered by the automated remedy finder (Table 1).

### 3.3. Medical Disclaimer

A total of 16 of the 74 possible complaints covered by the automated remedy finder included a medical disclaimer before proceeding to the questionnaire. This statement was directed at anyone selecting the target complaint regardless of that complaint’s severity in the individual. In comparison, in the live clinic cases selected, there was the possibility that while any complaint could warrant a referral to medical care, only one was deemed serious enough to pause homeopathic care and refer to a medical provider. Live practitioners saw cases of COVID-19 (10 cases), traumatic injuries (3 cases), and allergic reactions (1 case) in which homeopathic care was not paused. For COVID-19 cases, clinicians had access to a red flag guide developed by a licensed medical practitioner to assess when the client needed to seek immediate medical care.

The medical disclaimer was worded as follows: People with the condition you have indicated should seek medical attention as soon as possible. Please ensure that medical help has already been administered or has been summoned before proceeding to search for a remedy. A homeopathic remedy may be helpful on the way to the hospital, but this tool is no substitute for trained, experienced, in-person medical assistance.

### 3.4. Overall Remedy Match Rates (Live Practitioner vs. Automated Online Remedy Finder)

Symptoms from the 100 clinic cases reviewed fell into 22 of the 74 complaint categories covered by the automated remedy finder (see Appendix A). In the 100 cases compared, the automated online remedy finder included the AHE practitioner recommended remedy 59% of the time (see Table 2). There was a wide variance in the remedy match rate according to the complaint. The highest remedy match rate with multiple cases were COVID-19 and ear infections, each of which had an 80% match rate and one case in which there was a top match with the live practitioner. There were more COVID-19 cases than ear infections overall (10 cases vs. 5 cases). The lowest match rate with multiple cases was influenza, with no successful remedy matches in the 11 cases analyzed (see Table 2). The highest remedy match rates in individual case examples were seen in kidney stones, nausea of pregnancy, and sprains/strains, where there was a 100% match rate between the online remedy finder and the live practitioner.

Of the cases seen by the AHE clinic practitioners, 29 (23.2%) cases included a cough. This was more than any other complaint with a remedy match in 14 cases, or 48.3% for coughs overall (see Table 2). Common colds were also frequent, with 21 cases, or 16.8%, with a remedy match in 12 cases, or 57.1% overall. The next most frequent complaints were sore throat/hoarseness (16 cases or 12.8%), influenza (11 cases or 8.8%), COVID-19 (10 cases or 8%), headaches (6 cases or 4.8%), ear infections (5 cases or 4%), and conjunctivitis (4 cases or 3.2%). Less frequent but still occurring in more than one case were nausea and vomiting, traumatic injury, and urinary tract infections (3 cases of each or 2.4%), and dental complications, dizziness, traumatic injuries and teething (2 cases each or 1.6%). There were only single cases of allergic reaction, contact dermatitis, diarrhea, impetigo, insomnia, kidney stone, nausea of pregnancy, and sprains/strains, of which each single case represented 0.8% of the 125 total complaints analyzed.

### 3.5. Accuracy of Online Remedy Finder vs. Live Practitioner Recommendations

The online remedy finder provides the user a list of remedy recommendations in order of priority after the user has answered the algorithm-driven questions associated with the specific complaint. It was found that of the 100 cases compared there were 37 occasions in which the AHE clinic recommended remedy was among the top three online recommended remedies (see Table 2 and Table 3), including 17 cases in which the first remedy selected by the AHE Clinic and the online remedy finder coincided (see Table 2 and Table 3).

There were 17 occasions that the AHE clinic practitioner remedy recommendation was the same as the top recommended remedy in the online remedy finder. Of these 17 instances, drilling deeper into the presenting complaints in these cases, it was found that 6 of those cases related to coughs, 3 related to common colds, 2 related to sore throats and 1 instance each was found for COVID-19, ear infections, headaches, UTI’s, nausea and sprains (see Table 2 and Table 3).

Coughs generated the highest number of cases in which the top remedy recommendation from the online remedy finder algorithm matched the practitioner-recommended remedy, a total of six cases (see Table 1 and Table 3). Coughs were also the most frequently analyzed complaint in this study, with 29 cases overall, so when compared against the total number of cough cases, the top matches totaled only 23.2% (see Table 1). A more detailed view of cough cases is outlined in Appendix D.

Six of the 17 top remedy matches were the only top matches in their complaint category: COVID-19, ear infection, headache, nausea of pregnancy, strain/sprain, and UTI (See Table 1). Except for COVID-19, a complaint associated with 10 cases in total, the remaining complaints yielding top remedy matches consisted of six or fewer cases analyzed overall (see Table 1).

Two of the complaints that resulted in a top remedy match with the online remedy finder algorithm—nausea of pregnancy and sprains and strains—were the only cases in their respective complaint categories, resulting in a 100% overall match rate for the respective complaint categories. There were no complaints for which there were multiple case examples that resulted in a similarly perfect match rate (see Table 2).

Cases that resulted in a top remedy match did so with different homeopathic remedies, including cases in which different remedies were recommended for the same complaint (particularly coughs, the most frequent complaint seen in the study) as well as the same remedy being used for different complaints (see Table 3).

Cases that resulted in a top remedy match also did so with varying numbers of remedies recommended in total, including for the same complaint. For example, there were between 3 and 12 remedies recommended for cough cases depending on the answers provided in the questionnaire (See Table 4). Case # 55, a COVID-19 case that yielded *Arnica* as a top recommendation, was the first of 16 remedies recommended in total (see Table 4).

## 4. Discussion

### 4.1. Remedy Overlap/Case Management

The findings of this study show that the purpose-built homeopathic remedy finder is not a one-to-one replacement for a live practitioner. There is frequently an overlap between algorithm-generated acute remedy recommendations as well as general guidance provided on important issues such as how to take a homeopathic remedy (see Appendix B) and when to seek a licensed health care provider. However, in 41% of the cases analyzed, there was no remedy overlap at all, even with multiple algorithm-generated remedy recommendations. In addition, there is no guidance on situations that often arise in acute case management, such as how to manage an aggravation of symptoms after a potentially good remedy is given in too strong a potency or how to maintain healing momentum when the indicated remedy is still needed but its effectiveness stalls. While there were 17 out of 100 cases in which the practitioner-recommended remedy was the top suggestion by the automated remedy finder, depending on the complaint there were anywhere between 1 and 20 remedy recommendations. This has the practical effect of outsourcing the work of differentiating potential remedies to the sick individual. Although each remedy possibility is accompanied by a short description of the conditions under which it may be helpful for the given complaint, this is a potentially burdensome task, particularly when someone is ill.

### 4.2. Validation of the Tools

This study also highlights the challenge and importance of validating remedy recommendations against real-world cases. The automated remedy finder we compared covers 74 acute complaints, and the sample set of 100 randomly selected cases from the AHE clinic covered only 22 of them, clustering mostly around coughs, colds, sore throats, influenza, and COVID-19. A brief analysis of coughs—the most common complaint investigated in this study—can be found in Appendix C. Finding real-world data to validate just the automated remedy finder’s algorithm would require far more cases than this small study. As purpose-built tools become more developed, this validation will be needed to add credibility to remedy recommendations. However, the collection of the data needed to improve the model will ultimately require both the consent and participation of the users, and the full buy-in for robust validation processes by the tools’ creators and owners. While the automated remedy finder does have a way for users to report on whether the remedy they took was supportive, this is an ‘optional’ feature of the software we investigated, and it is unclear how robustly users provide feedback necessary to “train” the model. No such mechanism appears to exist for commercially available large language models (LLMs), which can be used for free by anyone interested in obtaining homeopathic remedy recommendations. We know little about this phenomenon, but we believe it to be an important area of future research.

The 59% overall remedy match rate found during the study is due to many factors, including the limitations of the study design—however, there are likely other factors as well. For example, during the study, many answers to the questions asked were “not applicable” or “none of the above” or “I don’t know.” While this phenomenon was no doubt due in part to the fact that the researchers were working from case notes, a robust validation process could identify and minimize low impact questions from live users (i.e., targeted questions unlikely to elicit a positive answer that points to a certain remedy or group of remedies) and identify and maximize high impact questions from live users (i.e., targeted questions that are likely to lead to a positive answer that points to a certain remedy or set of remedies). This is, after all, the promise of AI—a sophisticated algorithm that “learns” based on information provided to it.

### 4.3. Empowerment of Users

The automated remedy finder was designed to handle acute, self-limiting complaints only. This may ultimately be the best-use scenario for AI tools in the field of homeopathy, following on a long American family tradition of home prescribing [1]. Many of the more sophisticated interview techniques and case management skills required for the successful understanding and management of chronic cases are less critical in the context of acute complaints, so there is a greater potential to play on the strengths of automation without risking the downsides of outsourcing complex care to automated tools that are simply not up to the task. However, the guardrails in place with automated remedy finders are not present in commercially available LLMs, which will provide remedy recommendations on both straightforward acute complaints as well as more complex and serious chronic complaints, such as suicidal depression. The potential risks of outsourcing health care to automated models, a phenomenon which is highly possible given growing public dissatisfaction with the conventional medical system [22,23], calls for self-empowerment in health care [24,25], and mutual informal health care support from social media groups [26,27] with its unknown impacts and consequences needs to be researched in order to be more fully understood.

### 4.4. Limitations

There were a number of limitations in the study design that likely affected its outcome, the most important of which was that the symptom information entered into the automated questionnaire came from written case notes, not directly from the symptomatic individual. This frequently led to a situation in which researchers had to enter null answers, such as “I don’t know,” “Not applicable,” or “No” as an answer in the automated questionnaire because there was no information in the case notes relating to certain questions. A good example of this was a multiple choice question in the Sore throat/Hoarseness category, which contained the question, “Are the tonsils affected? (Look on either side of the tongue in the back of the mouth) (choose all that apply).” Unless there was specific information in the case notes about this symptom (which a symptomatic individual would have easily been able to answer by looking at their own throat), researchers had to enter a null answer, which in this case was “No, the tonsils are not visible or appear as they normally do”.

This structural limitation likely decreased the amount of relevant information fed into the automated questionnaire, which in turn may have lowered the match rate, albeit by an unknown amount. In the Sore throat/hoarseness category, for example, the positive remedy match rate was 10/16, or 62.5%, with 9/16 (56%) of the remedy matches appearing in the top three results from the algorithm. However, 63% of the answers entered into the algorithm were negative/null answers. While a symptomatic individual would have also entered negative and null answers, there would almost certainly have been a lower percentage. The question of how to optimize an algorithm to minimize low impact questions (i.e., questions more likely to result in a negative/null answer) is an important but separate question beyond the scope of this paper. An initial investigation into this general phenomenon is discussed with reference to the questionnaire for coughs in Appendix C, but the results were inconclusive because the sample numbers were so low.

Another limitation of the study is the underlying assumption that live practitioners consistently gave good remedy recommendations that ameliorated the complaint. While the overall results of the clinic were positive—81.6% of the cases resulted in “resolved” or “much better” (see Appendix D), these ratings were collected at the end of the encounter with clients, which often involved more than one remedy recommendation throughout the course of treatment. To more accurately determine the effect of the first remedy recommendation (i.e., the recommendation used to compare against the algorithm recommendations), a more focused study would be required that excluded cases in which there were not clear improvements with the first recommended remedy.

An additional limitation relates to evaluation of criteria for inclusion or exclusion in the study. Each researcher responsible for evaluation of inclusion criteria did so independently. Inclusion/exclusion decisions were then made based on a comparison of researcher decisions. In cases where there was a conflict of decision, particular care was taken to avoid speculation about what led to certain remedy selection. Agreement was reached on case inclusion/exclusion before continuing with further analysis. Researchers had access to case notes that went beyond the live practitioners’ initial remedy recommendations, which could have skewed the results if included. However, this adherence to providing answers based only on the text in the case notes from the initial case notes likely resulted in a higher percentage of negative/null answers, which may have decreased the likelihood of remedy matches.

This study was limited by the number of cases used in the study. In order to evaluate the algorithm for any of the covered complaints, a significantly larger number of cases would need to be analyzed. The resulting remedy match rates, when categorized by complaint, vary widely according to how many cases were run through the algorithm (see Table 2). The complaint with the most cases—coughs—resulted in a remedy match in 16 of 29 cases tested (55%).

## 5. Conclusions

Limitations aside, this study is the first to compare the recommendations of live homeopathy practitioners to an online automated remedy finder.

Overall, the automated remedy finder provided a clear framework for acute prescribing by asking targeted questions for a limited set of acute complaints that can safely be treated at home, with basic guidance on how to take the remedy and built-in warnings for when to seek care from a licensed health care provider. The automated remedy finder was not able to go beyond basic recommendations in these areas, however, so commonly encountered situations in ‘real-world’ acute case management—such as remedy aggravations and potency stalls—were not covered.

The primary aim of this study, which was to compare remedy recommendations between an automated remedy finder and a live practitioner, showed significant gaps between live and automated remedy recommendations. Even in cases of remedy overlap, the final remedy differentiation is left to the user, who is asked to analyze his/her symptoms against the characteristics of anywhere between 1 and 20 remedy recommendations. This finding—let alone the additional skills necessary to successfully manage a case once a remedy is chosen—demonstrates that at present there is no equivalent substitute for a guided homeopathic interview from a live practitioner that results in a single remedy recommendation that can be altered as needed depending on the remedy response.

There is a potential role for more advanced AI tools to be employed in homeopathic prescribing. Emerging AI technology has the potential to compile feedback from real-world remedy responses, providing for the possibility of validation. The potential of emerging AI technology to assist in sifting through large amounts of literature and to “learn” based on feedback from remedy recommendations provides the possibility for evidence-based prescribing that could move the profession forward, most likely in the context of acute complaints. As a consequence, there are innumerable further future research investigations that emerge. Replicating the aim of this research using large language models is an obvious next step. Investigating the challenges of training of LLMs is another. The privacy concerns of using real world health data are a clear challenge.

The investigators did not assume that the online remedy finder used for this study is comparable to other commercially available remedy finders. Future investigations could reveal significant differences between different commercial products. However, the structural questions about the nature of complaints covered, how to elicit valuable answers to questions in a way that points to potentially supportive remedies, how to incorporate feedback to improve model accuracy, and patient safety/case management limitations would have been applicable to any model investigated. The purpose of this study, therefore, was not to evaluate a specific remedy finder but rather to explore the phenomenon of AI in homeopathy and begin a discussion.

As artificial intelligence tools continue to evolve, there are important considerations for homeopathic prescribing. While there are exciting possibilities, it will be important to find ways to take advantage of the things that AI tools can do well without sacrificing the things that only homeopathic practitioners are uniquely positioned to do.

## Figures and Tables

**Table 1:** Cases excluded from analysis because MYCaW complaints were not covered by automated remedy finder.

	MYCaW Symptom 1	MYCaW Symptom 2
96	Anxiety	Spiraling
52	Fatigue	Inconsolable night crying
60	Back pain	Impediment to movement
4	Pain in jaw on left after taking Causticum on own	Lower jaw misaligned
136	Stomach pain	Crummy feeling
198	Lost appetite	Mood
5	Rash	Constipation
275	Pulling	Cramping
180 *	Appearance of rash	Itching
117	Lethargy	Whining and touchiness
306 **	Frequent urination	Bowel movements
35	Blister	Pain

* Researchers checked against the following listed complaints but did not see a match for the following case: allergic reactions, diaper rash, and contact dermatitis. A subsequent case (# 29) with similar symptoms (“Face eruptions” and “Burning and itching”) did match with the remedy finder complaint category for “Impetigo”). ** MYCaW symptom of “frequent urination” was a possible match for complaint category for “UTI” but case notes show that this was another type of case.

**Table 2:** Comparison of AHE clinic practitioner remedy recommendations to the automated online remedy finder recommendations (*n* = 125 *).

	Number of Cases Analyzed	% of Cases Overall	RX Match	% of RX Matches in Complaint Category	Top 3 RX Matches	% of Top 3 RX Matches Overall	# 1 RX Matches	% of # 1 Matches Overall
Cough	29	23.2%	14	48.3%	9	31.0%	6	20.7%
Common Cold	21	16.8%	12	57.1%	6	28.6%	3	14.3%
Sore throat/hoarseness	16	12.8%	9	56.3%	7	43.8%	2	12.5%
Influenza	11	8.8%	0	0.0%	0	0.0%	0	0.0%
COVID-19	10	8.0%	8	80.0%	3	30.0%	1	10.0%
Headache	6	4.8%	3	50.0%	3	50.0%	1	16.7%
Ear infection	5	4.0%	4	80.0%	2	40.0%	1	20.0%
Conjunctivitis	4	3.2%	1	25.0%	1	25.0%	0	0.0%
Nausea and vomiting	3	2.4%	1	33.3%	0	0.0%	0	0.0%
Traumatic Injury	3	2.4%	2	66.7%	1	33.3%	0	0.0%
Urinary tract infection	3	2.4%	1	33.3%	1	33.3%	1	33.3%
Dental complications	2	1.6%	0	0.0%	0	0.0%	0	0.0%
Dizziness	2	1.6%	0	0.0%	0	0.0%	0	0.0%
Teething	2	1.6%	1	50.0%	1	50.0%	0	0.0%
Allergic reaction	1	0.8%	0	0.0%	0	0.0%	0	0.0%
Contact dermatitis	1	0.8%	0	0.0%	0	0.0%	0	0.0%
Diarrhea	1	0.8%	0	0.0%	0	0.0%	0	0.0%
Impetigo	1	0.8%	0	0.0%	0	0.0%	0	0.0%
Insomnia	1	0.8%	0	0.0%	0	0.0%	0	0.0%
Kidney stone	1	0.8%	1	100.0%	1	100.0%	0	0.0%
Nausea of pregnancy	1	0.8%	1	100.0%	1	100.0%	1	100.0%
Strains/Sprains	1	0.8%	1	100.0%	1	100.0%	1	100.0%
TOTAL	125 *	100.0%	59		37		17	

* If there was no remedy match on MYCaW Symptom 1, MYCaW Symptom 2 was used. # is referred as ‘number’.

**Table 3:** Number of AHE Clinic practitioner remedy recommendations that appeared in the top 3 list of recommended remedies in the online remedy finder (*n* = 37).

	Number	Percentage
1st remedy match	17	46%
2nd remedy match	7	19%
3rd remedy match	13	35%

**Table 4:** Presenting Complaints of cases in which there was a direct match between AHE clinic practitioner recommendation and a top remedy match using the online remedy finder (*n* = 17).

Complaint	Case #	# 1 RX	Total Online Remedy Finder Remedies Recommended
Cough	32	*Phosphorus*	11
Cough	48	*Drosera*	6
Cough	222	*Pulsatilla*	3
Cough	124	*Drosera*	10
Cough	178	*Spongia*	7
Cough	174	*Drosera*	12
Headache	50	*Belladonna*	7
Common cold	14	*Pulsatilla*	6
Common cold	20	*Kali bichromium*	7
Common cold	279	*Pulsatilla*	7
COVID-19	55	*Arsenicum*	16
Nausea of pregnancy	139	*Sepia*	4
Sore throat/hoarseness	241	*Lachesis*	5
Sore throat/hoarseness	169	*Mercurius*	7
Urinary tract infection	76	*Cantharis*	7
Ear infection	93	*Belladonna*	8
Sprains/strains	223	*Rhus toxicodendron*	4

# is referred as ‘number’.

## Data Availability

The data presented in this study are available on request from the corresponding author due to patient privacy restrictions.

## Abbreviations

The following abbreviations are used in this manuscript:

AHE	Academy of Homeopathy Education
LLM	Large language models

## Appendix A. Complaints Covered by Automated Remedy Finder

Symptoms from the 100 clinic cases reviewed fell into 22 of the 74 complaint categories covered by the automated remedy finder (each case had 2 possible symptoms, and a total of 125 symptoms were run through the automated remedy finder):

1. Cough (29 cases);
2. Common cold (21 cases);
3. Sore throat/hoarseness (16 cases);
4. Influenza (11 cases);
5. COVID-19 (10 cases);
6. Headache (6 cases);
7. Ear infection (5 cases);
8. Conjunctivitis (4 cases);
9. Nausea/vomiting (3 cases);
10. Traumatic injuries (3 cases);
11. UTI (3 cases);
12. Dental complications (2 cases);
13. Dizziness (2 cases);
14. Teething (2 cases);
15. Allergic reaction (1 case);
16. Contact dermatitis (1 case);
17. Diarrhea (1 case);
18. Impetigo (1 case);
19. Insomnia (1 case);
20. Kidney stones (1 case);
21. Nausea of pregnancy (1 case);
22. Strains and sprains (1 case).

The following 52 complaints were covered by the remedy finder but not used in the analysis of the 100 cases:

1. Abdominal bloating/flatulence
2. Allergic rhinitis
3. Anticipatory anxiety
4. Bell’s palsy
5. Bone fracture
6. Brooding
7. Bruising
8. Burns
9. Bursitis
10. Chest pain
11. Chickenpox
12. Circulatory shock
13. Cold sores
14. Colic
15. Croup
16. Crush injuries
17. Diaper rash
18. Drowning
19. Ear pain, external
20. Eruptive fevers
21. Fainting
22. Fright or panic
23. Frostbite
24. Gout
25. Grief
26. Head injury/concussion
27. Heartbreak
28. Heartburn
29. Hemorrhoids
30. Herpes genitalis
31. Hiccough (in children)
32. Hot flashes
33. Insect bites/puncture wounds
34. Intercostal neuralgia
35. Laryngitis
36. Mastitis
37. Menstrual cramping
38. Motion sickness
39. Mouth sores
40. Mumps
41. Nosebleed
42. Pleurisy
43. Poisoning (food or alcohol)
44. Post-shingles pain
45. Sciatica
46. Separation anxiety
47. Singles (Herpes Zoster)
48. Styes
49. Toothache
50. Vaginal discharge
51. Warts
52. Wounds to skin

## Appendix B. Posology Instructions from Purpose-Built Online Homeopathic Remedy Finder [28]

### Appendix B.1. How to Give a Remedy

Homœopathic remedies come in different prepared strengths called “potencies.” The C potencies are the most common. Stores often carry two racks of remedies, one 6C and one 30C (Some remedies, like Arnica montana, may be available in a separate rack at the higher 200C potency).

In most acute illnesses, any potency of the correct remedy will be effective. A lower potency (6C, for example) may need to be repeated more often than a higher potency.

If you have a choice of potencies:

- Use a 6C potency when the symptoms are mild, if the person is very sensitive (i.e., has allergies) or if you are not positive of the match between the remedy description and the person’s symptoms.
- Use a 30C potency when the symptoms are a bit clearer or stronger, or if there is a good match between the remedy description and the person’s symptoms.
- Use a 200C potency (where available) when the symptoms are strong and the remedy description matches the person’s symptoms perfectly.

Wondering how many pellets of a remedy to take? Just follow the instructions we give you.

### Appendix B.2. Managing the Dose

Wondering when to repeat or change a remedy? Click here (https://www.homeopathichousecall.com/resource/manage-a-remedy accessed on 8 July 2025).

### Appendix B.3. Managing a Remedy Dose

#### Appendix B.3.1. Repeating an Effective Dose

The remedy worked! Yay! Now, how often do I give it? The universal rule is this: As long as improvement continues, do not repeat the remedy. When improvement stalls, as long as the fundamental character of the symptoms have not changed (only the intensity), that is the time to give another dose. The closer a person gets to full recovery, the less often they should need to take the remedy.

#### Appendix B.3.2. Nothing Happened, What Do I Do?

If you observed no change in the person’s symptoms after one dose of the remedy, and you have allowed a reasonable amount of time for the remedy to act, you probably want to give one more dose of that remedy before changing remedies. If nothing happens after two doses, consider another remedy from your results.

#### Appendix B.3.3. When to Change a Remedy

If a remedy is not effective after two doses, there is probably a better match for the person’s symptoms. Likewise, if the fundamental character of the symptoms changes, it is time to look for a new remedy based on the new symptoms.

## Appendix C. Analysis of Cough Complaints—Comparison Between Purpose-Built Online Homeopathic Remedy Finder and Live HHN Practitioners

Coughs were the most frequently featured complaint, representing 29 of the 125 complaints run through the HH algorithm.

14/29 (48%) of the cases including a cough were a match with HHN recommended remedies.

9/29 (31%) of the HH matches put the HHN recommended remedy among the top 3 HH rx choices,

3/29 (10%) of the HH matches put the HHN-recommended remedy among the bottom 3 HH rx choices.

Every cough questionnaire included the following questions:

1. What seems to trigger the cough?
2. How does the cough sound? (choose all that apply)
3. Do any of the following happen with the cough? (choose all that apply)
4. Is the person coughing up phlegm?
5. If the person is an infant, is the child having difficulty nursing because of the mucus blocking the nose?
6. Is the cough causing pain anywhere? (choose all that apply)
7. If the person is coughing up phlegm, do any of these phrases describe the phlegm?
8. Which of the following statements, if any, are true of the person’s cough? (choose all that apply)
9. Does the person want to blow their nose after coughing?
10. Did the cough start after an asthma attack?
11. Did the cough appear since a cold or other respiratory infection?
12. Does the person hold their chest when they cough?
13. What time of day, if any, is the cough worse? (choose all that apply)
14. Which of the following circumstances, if any, make the cough better? (choose all that apply)

Depending on answers to previous questions, the questionnaire sometimes included the following questions:

1. (if dry cough is selected) Is the cough mainly dry at night and loose during the day?
2. (if loose cough is selected) Is the cough mainly loose in the morning?
3. (if the cough is causing pain) Is it a burning pain?
4. (if the cough is occurring in fits and attacks) Do the attacks or fits come rapidly after one another?
5. (If the cough is worse in the morning) Is the cough worse in mornings and at 11pm?
6. (if the cough is worse lying down) Is the cough only worse when lying on the left side?

Case # 280 triggered the highest number of questions (18) but did not have a remedy match with the live practitioner. Ten of the 18 questions asked in this case resulted in negative or null answers, which was 55.56%.

Overall, the negative/null answers for coughs averaged 67.34%.

Cases that successfully matched a remedy with a practitioner had a lower percentage of negative/null answers: 64.5%.

Cases that had a top remedy match with a practitioner averaged 68.13% null answers

## Appendix D. Table of 100 Cases—Comparison Between Purpose-Built Online Homeopathic Remedy Finder and Live HHN Practitioners

**Table:**

Randomized Case Number	HH 1st Complaint Used	HHN RX	RX Match?	HH 2nd Complaint Used	2nd Complaint RX Match?	Remedy Response Scale Change Intake/Case Closed
32	Cough	Phos	yes			resolved
8	Cough	Puls	yes			resolved
109	headache	Bry	no	COVID-19	yes	resolved
273	Sore throat/ hoarseness	Lach	yes			much better
169	N/A	Merc		sore throat/hoarseness	Yes	
258	N/A	Puls		cough	Yes	resolved
48	cough	Dros	yes			resolved
108	common cold	Kali-bi	yes			much better
50	headache	Bell	yes			somewhat better
64	COVID-19	Phos	Yes			much better
47	COVID-19	Puls	yes			much better
118	sore throat/ hoarseness	Lyc	yes			resolved
159	headache	Bry	yes			resolved
261	cough	Phos	yes			resolved
190	cough	Bry	yes			resolved
86	common cold	Phos	no	cough	Yes	resolved
42	common cold	Nux-v	yes			resolved
222	cough	Puls	yes			somewhat better
124	cough	Dros	yes			somewhat better
14	common cold	Puls	yes			much better
152	ear infection	Puls	yes			much better
20	cough	Kali-bi	no	common cold	yes	resolved
296	cough	Puls	yes			much better
37	common cold	Nux-v	yes			much better
111	kidney stone	Berb	yes			unresolved
18	cough	Sulph	yes			resolved
206	influenza	Puls	no	common cold	yes	resolved
154	sore throat/ hoarseness	Lach	yes			
145	N/A	Cham	yes	teething	yes	much better
55	COVID-19	Ars	yes			much better
235	dizziness	Arn	no	traumatic injury	yes	somewhat better
216	common cold	Puls	yes			much better
189	traumatic injuries	Arn	yes			much better
132	common cold	Puls	yes			somewhat better
123	conjunctivitis	Puls	yes			much better
250	ear infection	Acon	yes			resolved
191	sore throat/ hoarseness	Hep	yes			resolved
66	COVID19	Sulph	yes			resolved
84	COVID19	Phos	yes			resolved
1	N/A	Ars		common cold	yes	resolved
139	nausea of pregnancy	Sep	yes			resolved
116	nausea and vomiting	Cocc	yes			somewhat better
241	sore throat/ hoarseness	Lach	yes			much better
238	ear infection	Merc	yes			resolved
7	COVID19	Bry	yes			much better
218	common cold	Puls	yes			no response
178	cough	Spong	yes			much better
93	influenza	Bell	no	ear infection	yes	much better
197	cough	Phos	yes			somewhat better
129	cough	Puls	no	Headache	yes	much better
283	sore throat/ hoarseness	Merc	yes			resolved
174	cough	Dros	yes			much better
51	influenza	Puls	no	COVID-19	yes	much better
223	traumatic injuries	Rhus-t	no	strains/sprains	yes	resolved
303	common cold	Lach	no	sore throat/hoarseness	yes	resolved
76	Urinary tract infection	Canth	yes			unresolved
279	sore throat	Puls	no	common cold	yes	resolved
166	influenza	Puls	no	common cold	yes	resolved
79	sore throat/ hoarseness	Lach	yes			resolved
10	Sore throat/ hoarseness	Calc-c	no	Headache	no	blank
115	Insomnia	Ip	no	cough	no	resolved
307	cough	Coc-c	no	N/A		no response
162	cough	Calc-c	no	N/A		resolved
274	urinary tract infection	Puls	no	N/A		somewhat better
271	N/A	Phos		Headache	no	resolved
133	dizziness	Nux-v	no	sore throat/ hoarseness	no	somewhat better
237	sore throat/ hoarseness	Ars	no	N/A		much better
246	nausea and vomiting	Phos	no	influenza	no	resolved
220	urinary tract infection	Puls	no	N/A		resolved
211	cough	Kali-c	no	common cold	no	much better
73	Sore throat/ hoarseness	Lyc	no	conjunctivitis	no	much better
290	conjunctivitis	Sil	no	N/A		much better
193	cough	Kali-c	no	N/A		resolved
184	Sore throat/ hoarseness	Kali-bi	no	N/A		much better
57	dental complications	Calen	no	N/A		somewhat better
278	COVID19	Bry	no	cough	no	much better
106	cough	Acon	no	teething	no	resolved
72	COVID19	Kali-c	no	common cold	no	much better
100	ear infection	Sil	no	N/A		resolved
134	N/A	Samb		cough	no	resolved
175	cough	Bell	no	influenza		resolved
286	N/A	Bell		allergic reaction	no	much better
243	common cold	Kali-c	no	N/A		somewhat better
77	N/A	Op	no	diarrhea	no	somewhat better
265	influenza	Puls	no	N/A		resolved
29	impetigo	Calc-c	no	N/A		much better
301	conjunctivitis	Bell	no	N/A		resolved
121	dental complications	Merc	no	N/A		resolved
49	influenza	Sil	no	common cold	no	resolved
251	cough	Sang	No	Client did not list 2nd symptom		resolved
83	N/A	Calc-c	no	cough	no	resolved
75	contact dermatitis	Apis	no	N/A		resolved
249	common cold	Sil	no	N/A		somewhat better
26	influenza	Nux-v	no	N/A		much better
207	N/A	Merc		influenza	no	resolved
23	sore throat/ hoarseness	Ars	no	N/A		resolved
280	cough	Puls	no	N/A		resolved
192	cough	Ferr-p	no	Client did not list 2nd symptom		resolved
22	common cold	Nat-m	no	N/A		resolved
112	influenza	Bell	no	common cold	no	resolved

“N/A” depict situations in which the client’s symptom was not covered by the HHC algorithm as a distinct complaint (e.g., “fatigue,” “fever,” “exhaustion,” “throat patches,” “irritability”) or identical to the other complaint (e.g., “urethra pain” and “urgency” both fell under the “urinary tract infection” complaint in HHC).
